# Utility of Constructive Interference in Steady-State Sequence in Detecting Thin Pituitary Stalk in Pituitary Stalk Interruption Syndrome

**DOI:** 10.7759/cureus.16105

**Published:** 2021-07-02

**Authors:** Sanjay M Khaladkar, Pranav Ajmera, Ragamayi Maramraju, Isha Kedia

**Affiliations:** 1 Radiodiagnosis, Dr. D. Y. Patil Medical College, Hospital and Research Centre, Pune, IND; 2 Radiology, Dr. D. Y. Patil Medical College, Hospital and Research Centre, Pune, IND; 3 Medicine, Dr. D. Y. Patil Medical College, Hospital and Research Centre, Pune, IND

**Keywords:** pituitary stalk interruption syndrome, pituitary stalk transection syndrome, infundibulum, ectopic posterior pituitary, hypopituitarism

## Abstract

Pituitary stalk interruption syndrome (PSIS) is a rare congenital anomaly, causing hypothalamic-pituitary malfunction. It is characterized by hypoplastic anterior pituitary, absent or thin infundibulum, and absent or ectopic posterior pituitary. Its early recognition is important in disease management. MRI plays a pivotal role in early diagnosis.

We report a case of a 13-year-old male child, presenting with stunting of growth and discrepancy between chronological and bone age of four years. A subsequent MRI revealed a small anterior pituitary, hypoplastic pituitary stalk, and an absence of visualization of the bright pituitary gland signal in the sella. The posterior pituitary gland was present ectopically in the midline along the floor of the third ventricle near the median eminence.

## Introduction

Pituitary stalk interruption syndrome (PSIS) is a rare congenital anomaly with an incidence of 0.5 per 1 lac live births. It has a male preponderance and causes hypothalamic-pituitary axis malfunction. It presents with short stature. Associated anomalies are corpus callosum dysgenesis, heterotopia, optic nerve hypoplasia, and olfactory nerve abnormalities. It has three components (triad): Hypoplastic anterior pituitary (98.3%), absent (98.3%)/hypoplastic (<1 mm) pituitary stalk/infundibulum, and ectopic (91.4%) or absent posterior pituitary gland/no eutopic posterior lobe [1]. Ectopic neurohypophysis was observed in the infundibular recess (60.4%) and in the hypothalamus (18.95%) [2]. Its early recognition and management of the disease can allow for an improvement in the growth stature of the patient [1,2]. It is also called the syndrome of interruption of the pituitary stalk (SIPS). It was first described in 1987 by Fujisawa et al. [3].

It can cause isolated growth hormone deficiency (IGHD) or combined pituitary hormone deficiency (CPHD). Usually, it results in the progressive onset of hormone deficiency finally leading to panhypopituitarism. The posterior pituitary function is usually maintained. Rarely, it can be affected depending on its position [4]. It has male predominance with a Male:Female sex ratio between 2:3 and 6.9:1.0, suggestive of X-linked inheritance [4-6].

Its prevalence as a cause of GH deficiency is around 4% [7]. Thyroid-stimulating hormone (TSH) deficiency, along with GH deficiency, is seen in 75.9% of cases, adrenocorticotropic hormone (ACTH) deficiency is seen in 67.5% of cases, and follicle-stimulating hormone/luteinizing hormone (FSH/LH) deficiency in 65.1% cases [7].

## Case presentation

**Figure 1:**
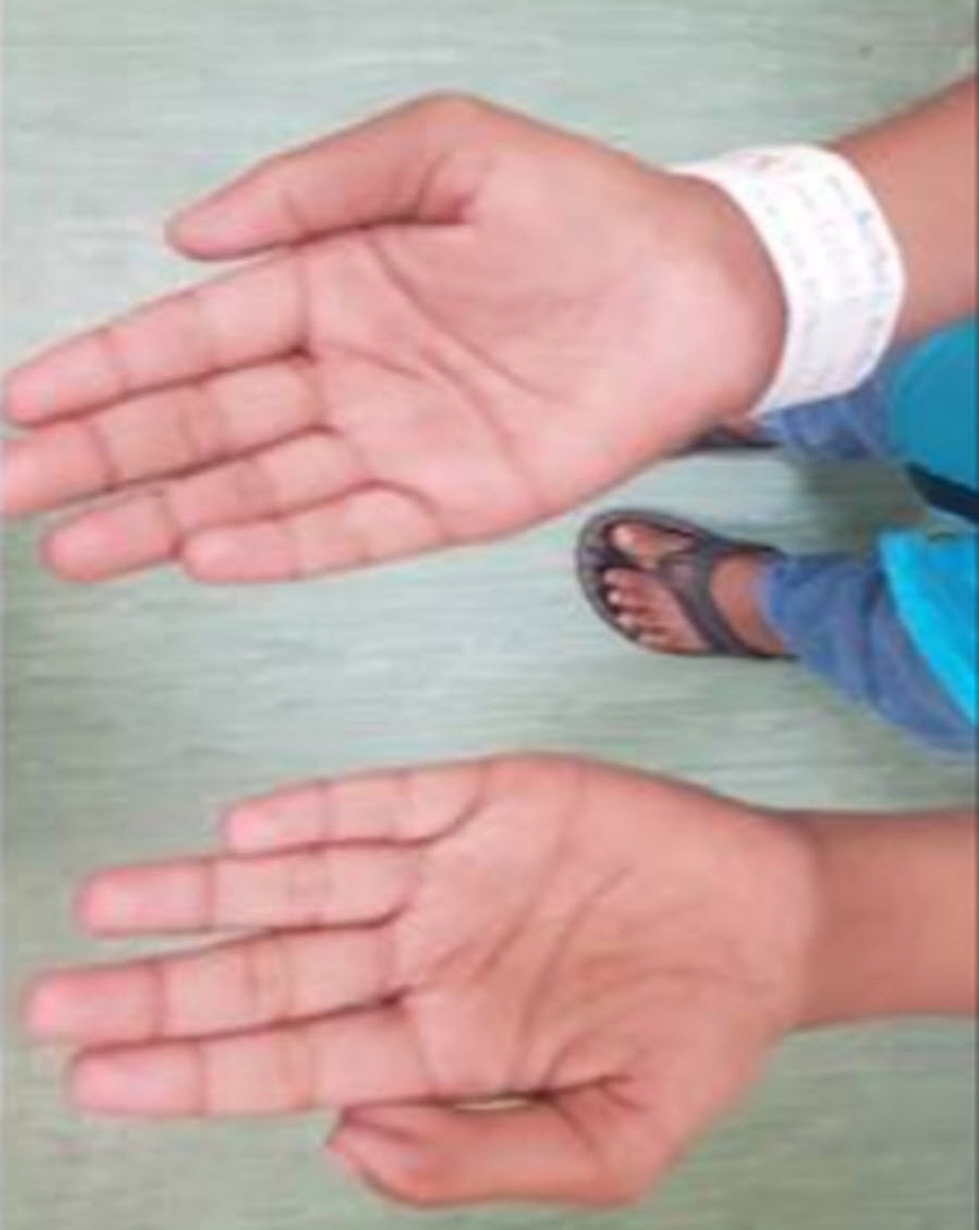
Image of bilateral hands demonstrating preaxial polydactyly in the left hand.

A 13-year-old male child presented with short stature. The child had globally delayed milestones and polydactyly in his left hand (Figure 1).

**Figure 2:**
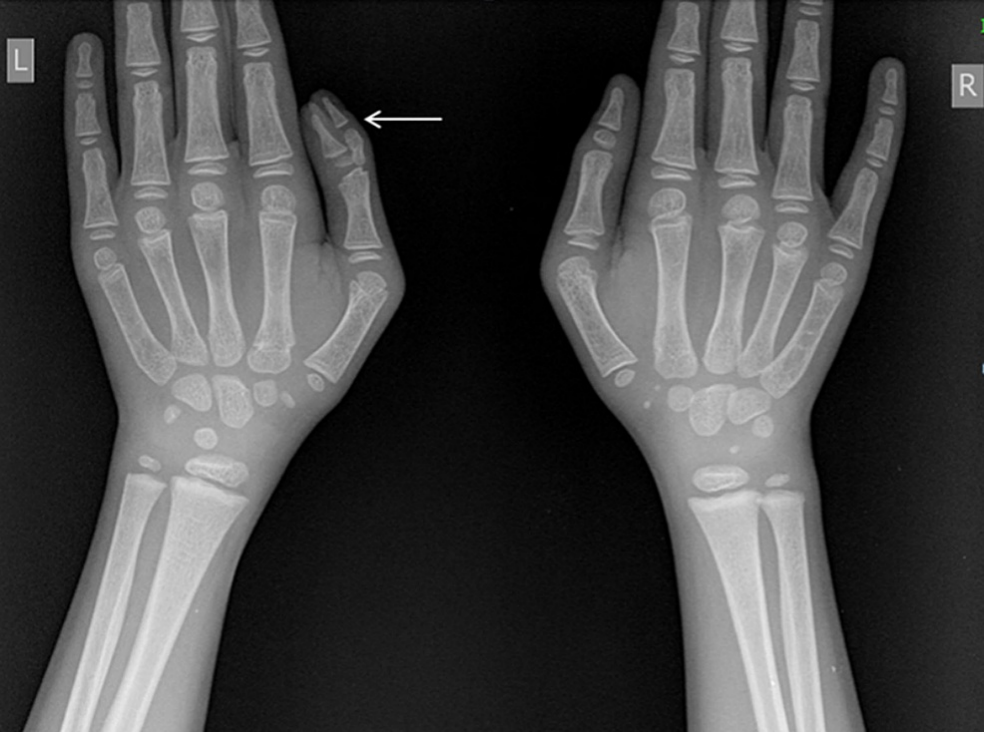
Radiograph of bilateral hands demonstrating polydactyly in the left thumb.

A detailed physical examination and anthropometry were performed, and it revealed that the height of the boy was lesser than the target height, when calculated after taking into account the height of the parents. Subsequently, a full panel of blood investigations was performed, which revealed hemoglobin = 9.2 g/dl (low), total leucocyte count= 4800/cubic mm (low), normal differential leukocyte count, and microcytic hypochromic anemia on the peripheral blood smear. He also underwent a detailed evaluation of the full panel of serum hormone levels, these revealed: LH <0.1 mIU/ml (N = <0.1-6 IU/ml); FSH <0.3 mIU/ml (borderline low, N = 0.3-10); T3 = 84 ng/dl (N = 80-120 ng/dl), T4 = 3.97 ugm/dl (low, since N = 4.2-12 ugm/dl), free T4 = 0.54 (low), TSH = 3.13 mIU/ml (N = 0.25-5.5 mIU/ml); cortisol = 7.9 (at 8 AM); human growth hormone (hGH) = 0.27 ng/ml (N = up to three in males); somatomedin-C = 75.7 ng/ml (significantly low, N = 183-850 ng/ml, for a 13-year-old child). Radiographs of the left hand with wrist and left elbow showed polydactyly and bone age of 5-8 years, implying a significant lag of bone age concerning the chronological age (Figure 2).

The child was diagnosed with GH deficiency-related stunting based on blood parameters, radiographs, and anthropometric findings.

**Figure 3:**
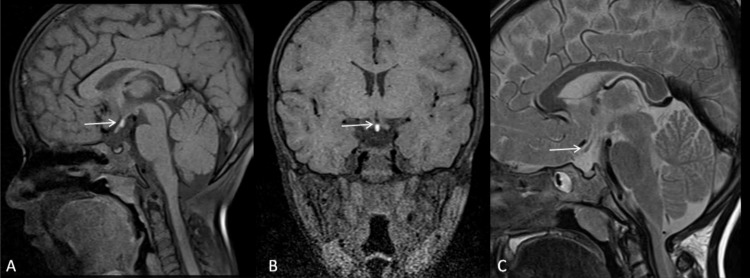
Sagittal (A) and coronal T1 (B), sagittal T2 (C): weighted MR images of pituitary fossa shows non-visualization of infundibulum, absence of bright spot of posterior pituitary at the normal site, ectopic posterior pituitary near the median eminence (marked by white arrow).

**Figure 4:**
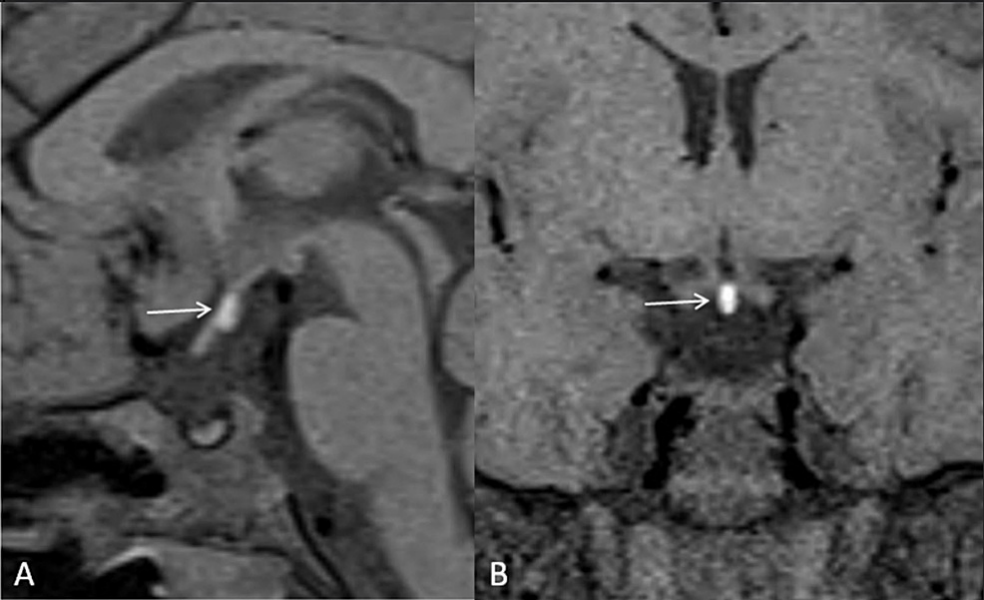
A magnified image of the sagittal (A) and coronal (B) T1W MR sections at the level of pituitary gland demonstrates small-sized anterior pituitary, non-visualization of infundibulum, absence of bright spot of posterior pituitary at the normal site, ectopic posterior pituitary near the median eminence (marked by white arrow).

**Figure 5:**
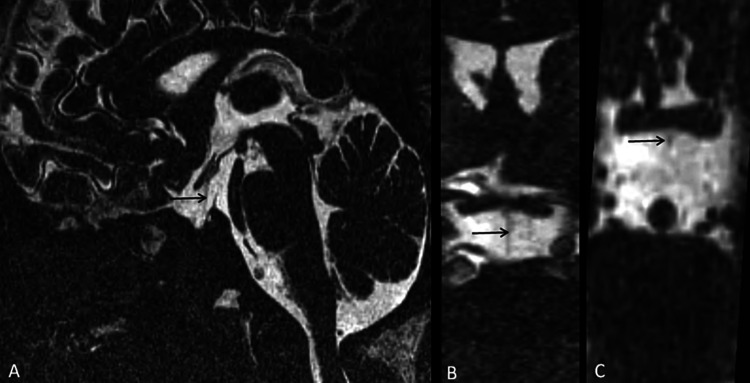
Sagittal (A), coronal (B), and axial (C) sections of CISS sequence on MR pituitary fossa demonstrates thin/hypoplastic infundibulum (marked by black arrow). CISS: Constructive interference in steady state.

MR brain (sagittal and coronal T1, T2WI, constructive interference in steady-state [CISS, sequence: sagittal, coronal, axial]) revealed a small anterior pituitary, hypoplastic/thin pituitary stalk, and absence of visualization of the bright posterior pituitary gland signal in the sella (Figures 3-5). The posterior pituitary gland was present ectopically in the midline along the floor of the third ventricle near the median eminence (Figures 3 and 4). These findings favored PSIS. The patient was subjected to regular GH therapy with 2 IU of GH daily.

## Discussion

The study protocol for the detection of PSIS is centered on the hypothalamus-pituitary axis. The acquisition protocol includes sagittal and coronal thin (2-3 mm thickness), Fast-spin echo ( FSE)-T1W, and FSE-T2W sequences. In a post-contrast study, the entire brain must be studied to rule out associated anomalies [8]. FAST1-MRI protocol is proposed to detect PSIS. It is performed without contrast administration, without sedation or anaesthesia, after six hours of water restriction. It includes only a sagittal T1 sequence with a 3.25 mins duration [9]. The new MRI sequence, CISS, can identify pituitary stalk in isolated pituitary hormone deficiency (IPHD) [10].

Abnormalities in PSIS include (A) An anomaly of the pituitary stalk (a) complete form: infundibulum is absent and not visualized. (b) incomplete form: thin/filiform pituitary stalk is visible on thin T2WI sequence or in post-contrast studies. (B) An anomaly of the posterior pituitary: normally the posterior pituitary is seen as a hyperintense signal on T1WI and enhances after contrast administration. In SIPS, it is ectopic in location, either at the infundibulum (50%) or in the hypothalamus. The abnormality can be limited to the ectopic posterior pituitary. (C) An anomaly of the anterior pituitary: anterior lobe is hypoplastic and the child presents with a height less than 2SD for age. Though it is normal in size, it can show delayed contrast enhancement in those having GH deficiency during the dynamic study. (D) Associated malformation of the midline: Arnold-Chiari malformation, total or partial agenesis of the corpus callosum, Dandy-Walker malformation, and basipharyngeal canal [8].

The definition of PSIS is widened over the last decade to include patients with (A) interrupted stalk with ectopic posterior pituitary and (B) interrupted stalk with absent posterior pituitary. Isolated missing posterior pituitary with normal pituitary stalk should not be confused as PSIS, which can be seen in 10% of the healthy population. Normally, the posterior pituitary bright spot is recognizable by the second month of life [11].

The clinical appearance of PSIS is determined by the patient's age at the time of diagnosis. It causes neonatal hypoglycemia, physiological/neonatal jaundice, cryptorchidism, and micropenis in newborns. It manifests itself in older children as short stature. It is important to diagnose PSIS as soon as possible. If untreated, it results in increased morbidity and mortality due to multiple pituitary hormone deficiencies (MPHD). It can result in insufficient weight at the onset of puberty with resultant short stature [8].

Individuals with PSIS usually present with anterior pituitary hormone deficiencies: GH (100%), gonadotropin (86.52%), corticotropin (75.28%), and thyrotropin (79.78%). This results in short stature, delayed puberty, hypoglycemia, central hypothyroidism, cryptorchidism, and micropenis [12].

The presence of neonatal hypoglycemia, hyperbilirubinemia, and microphallus need further follow-up to see whether they developed GH deficiency. A child with short stature and a deranged thyroid profile in the absence of thyroid antibodies needs neuroimaging to evaluate the pituitary gland [13].

Patients presenting with hypoglycemia, jaundice, seizures, cryptorchidism, and hypothyroidism in the neonatal period and growth retardation with pituitary hormone deficiencies should undergo neuroimaging to rule out PSIS [14]. Extra-pituitary manifestations in PSIS are commonly obscured, this includes midline defects in the brain, eyes, extra-cerebral abnormalities, including defects of heart, skin, and extremities [7]. PSIS is associated with congenital malformations in 20-50% of patients, particularly in the midline (for example, cleft lip, absence of diaphragm, hypoplasia of optic nerves, and bulging brain). This suggests that the disease-causing gene is linked to a gene involved in hypothalamic-pituitary development during embryonic development [8].

The exact pathogenesis is unknown. Theories of pathogenesis are (A) Genetic theory: abnormal HESX1, LHX-4, SOX-3, PROKR-2 genes are seen in few cases [8]. (B) There is an increased incidence of PSIS in those delivered by breach or C-section, or who have suffered from neonatal hypoxemia. However, no hypoxic damage to other structures sharing the same vascular supply is found. Breech delivery causes head deformation, leading to injury to the pituitary stalk and the pituitary. Head trauma can cause mechanical rupture or ischemic insult to the pituitary stalk as the stalk is stretched between the pituitary gland and mobile brain [10]. (C) Antenatal origin: as there is an association of PSIS with micropenis and cryptorchidism with familial or syndromic forms [8,10]. (D) Another theory is congenital hypoplasia or dysplasia of the pituitary gland, causing hypopituitarism. Failure of the neurohypophysis and its investing vascular plexus to descend completely into sella turcica occurs due to early fetal maldevelopment of the midline structures. Reduced hormone secretion occurs due to anterior lobe hypoplasia and dysfunction which results in breech presentation [10].

With the increasing use of MRI as a primary radiological modality in patients with panhypopituitarism, its incidence is on the rise with a mean age of 9.4 years +- 11.6 months at the time of diagnosis. PSIS is associated with isolated GH deficiency or multiple anterior pituitary hormone deficiencies. Thin pituitary stalk is associated with isolated GH deficiency. However, isolated GH deficiency may progress to multiple anterior pituitary hormone deficiencies in the second or fourth decade of life. This indicates the importance of close follow-up of the patient presenting with isolated GH deficiency [5]. PSIS is an important cause of the anterior pituitary deficiency. It can cause IGHD or multiple anterior pituitary hormone deficiencies: MPHD. GHD can be associated with an abnormality of other anterior pituitary hormones, posterior pituitary function is usually normal [8]. The status of the pituitary stalk is an indicator of hormone deficiency type. Absent pituitary stalk is associated with multiple deficiencies of the pituitary hormones. Thin pituitary stalk is associated with a deficiency of single GHs. Although the posterior pituitary is absent or abnormal, the incidence of diabetes insipidus is low. This is due to collateral flow between the hypothalamus and the pituitary. Pituitary stalk damage interrupts the drainage of antidiuretic hormone (ADH) and vasopressin from the hypothalamus to the posterior pituitary [15].

The ectopic posterior pituitary bright spot is usually formed at the medial eminence, rarely can be formed at the stalk. Visualization of an enhancing pituitary stalk on post-contrast images is suggestive of partial preservation of the hypothalamic-hypophyseal portal vessels with IGHD. Non-visualization of the pituitary stalk indicates the progression of the disease with MPHD. Usually, as the disease progresses from IPHD to MPHD, the stalk and adenohypophysis become smaller. Hence, thin stalk syndrome or pituitary stalk hypoplasia term should be preferred over the pituitary stalk transection syndrome [10]. The isolated ectopic posterior pituitary is the differential diagnosis of PSIS. It can be seen in apparently normal individuals and can be associated with idiopathic GH deficiency. Ectopic neurohypophysis functions relatively normally while adenohypophysis does not. This occurs due to an absent infundibulum that contains the hypothalamic-hypophyseal portal system communicating between the hypothalamus and the adenohypophysis. The ectopic posterior pituitary is 100% specific for GH deficiency in children with growth failure and has a 100% predictive value [9]. The presence or absence of PSIS on MRI may indicate that the IGHD is permanent or transient [9].

## Conclusions

Early diagnosis of PSIS is necessary by neuroimaging and hormonal assays in the detection of pituitary hormone deficiency, which can cause long-term morbidities. CISS sequence is extremely potent in looking at the pituitary stalk in such cases due to its superior resolution and thin cuts. Identification of deficient hormones and their replacement before fusion of growth plate can improve quality of life in patients with hypopituitarism. Regular follow-up is needed in cases of PSIS for the development of deficiencies of multiple pituitary hormones. A high index of suspicion of PSIS is needed in cases of GH deficiency with growth failure.
